# Centering Equity in Evidence‐Informed Decision Making: Theoretical and Practical Considerations

**DOI:** 10.1111/1468-0009.70002

**Published:** 2025-02-27

**Authors:** BOMI KIM HIRSCH, KIERSTEN FROBOM, GILLIAN GIGLIERANO, MICHAEL C. STEVENSON, MARJORY L. GIVENS

**Affiliations:** ^1^ University of Wisconsin Population Health Institute, University of Wisconsin–Madison

**Keywords:** health equity, evidence‐informed decision making, equity assessment

## Abstract

**Policy Points**

- The population health research field should develop a synthesized approach to evaluate evidence for an intervention's potential impact on equity.
- When empirical evidence is lacking, theory and frameworks should guide the equity assessments in four areas: 1) understanding historical context, meaning root causes of disparities and inequity; 2) understanding intervention design and intended beneficiaries; 3) understanding differential impact and intersectionality; and 4) understanding community context before implementing or scaling interventions.
- The synthesized approach of equity assessment better informs practitioners and policymakers in evidence‐based decision making to advance equity.

Over the past 50 years, population health researchers have made significant progress in clarifying the empirical and theoretical relationships between socioeconomic conditions and health disparities,^1^, ^2^, ^3^ particularly for social constructs such as race and ethnicity.^4^ Health disparities, defined as “differences in health or its determinants that adversely affect marginalized or excluded groups,” are the metric to measure progress toward health equity,^5^, ^6^ meaning “assurance of the conditions for optimal health for all people.”^7^ At the same time, policymakers and public health practitioners have invested in and implemented interventions (i.e., policies and programs) that have empirical evidence supporting their effectiveness in a process called evidence‐informed decision making (EIDM). EIDM refers broadly to a wide variety of approaches to find, assess, and implement relevant knowledge in decision making about social issues.^8^, ^9^ EIDM originated from evidence‐based medicine, a process popularized in the 1990s to improve clinical decision making by emphasizing systematic searches and evaluation of scientific literature and noting that “intuition, unsystematic clinical experience, and pathophysiologic rationale” were not sufficient for producing desirable outcomes.^10^ EIDM has been broadly adopted in many forms, including in population health, to improve decision making by practitioners and policymakers. Scientific or research evidence is often distinguished from tacit or colloquial evidence such as opinions, values, and habits and can be called “evidence based” or “evidence informed” to represent that research evidence is one of several factors influencing policymaking.^9^ EIDM—based on the process of searching, appraising, synthesizing, adapting, and implementing evidence into policies and practices^11^—improves effectiveness and efficiency of interventions, facilitates an efficient use of limited resources, and improves transparency of decision making.^9^ As our knowledge has deepened about what shapes health and results in health disparities, the data and evidence we use to support EIDM have not evolved and now largely lack the precision and focus needed to address health equity. In 2021, the World Health Organization named equity as an explicit principle to consider in EIDM and thus urged monitoring observable differences in health to identify groups at risk^9^—meaning at risk for health inequity. The US Centers of Disease Control and Prevention (CDC) also launched its Cultivate, Optimize, Reinforce, and Enhance (CORE) Commitment to Health Equity framework in 2021, which marked the CDC's first agencywide health equity strategy.^12^ Recent research encourages broadening focus beyond health disparities to their structural causes and corresponding structural interventions,^4^, ^13^ including scholar Camara Phyllis Jones, who notes, “Achieving health equity requires valuing all individuals and populations equally, recognizing and rectifying historical injustice, and providing resources according to need.”^7^

Despite increased calls for equity to be considered in EIDM,^14^, ^15^, ^16^ there is still not consensus about how equity should be defined and evaluated in the EIDM process. Also still missing is empirical evidence regarding interventions’ implications for health equity, which would guide EIDM. For example, a systematic review of food and nutrition policies and health outcomes for Indigenous groups in Western colonized countries indicated there were evidence gaps around the effectiveness of population‐level policies on health outcomes for Indigenous people.^17^ A study of peer‐reviewed literature on the effectiveness of active transportation interventions found that most studies are not considering equity in their design, analysis, or discussion.^18^ Other large reviews reveal similar missing evidence about the consideration of intervention impact on diverse populations in the existing body of research. A study of 140 systematic reviews of population‐level interventions for adolescent health found that only 30% reported on differences in health outcomes between groups with higher and lower socioeconomic status.^19^ This is further underscored in a descriptive analysis of 583 US‐based programs with evaluations in the Blueprints for Healthy Youth Development registry, which found that nearly a quarter of studies did not report race of program participants and about 70% did not report economic disadvantage status.^20^ These studies highlight critical gaps and issues in the funding, research agenda setting, methods, and population selection most relevant to EIDM today and mean that traditional approaches to assess an intervention's effectiveness, based on empirical evidence alone, are inadequate to understand implications for equity.

This paper builds on calls to action for EIDM to center health equity.^9^, ^18^, ^20^, ^21^ We recommend that the population health research field should develop a synthesized approach to assess available evidence for its potential impact on equity and disparities. We also call for continued investment in research and methods that uncover health disparities and the structured power imbalances that maintain them. A synthesized approach would combine findings from health disparity and health equity research into a framework that guides analysis of evidence for equity impacts. When empirical evidence is lacking, theory and conceptual models should guide the equity assessments. First, we summarize critiques of EIDM that can help account for why a synthesized approach does not currently exist. Next, we offer several theoretical and practical recommendations with which population health could begin to structure a synthesized approach, organized in four areas: 1) understanding historical context, meaning root causes of disparities and inequity; 2) understanding intervention design and intended beneficiaries; 3) understanding differential impact and intersectionality; and 4) understanding community context before implementing or scaling interventions. Finally, we reflect on how the field can employ EIDM to advance equity even as we simultaneously work to improve the evidence base. We note the unique role that evidence clearinghouses can play in EIDM to advance equity and acknowledge the highly contested and political challenges to evidence‐informed change in this contemporary era.

## Critiques of EIDM

A synthesized approach assessing interventions’ impacts on equity and disparities may not yet exist because EIDM and its related field, evidence‐informed policymaking (EIPM), lack critical analysis of the epistemologies behind most current research, which informs what evidence emerges and how it is used. We believe that EIDM should acknowledge implicit assumptions and values in the existing body of evidence to better address health equity.

With relation to equity, evidence generation is an often‐critiqued prerequisite in the EIDM process. Scholars note that the paradigm underpinning research is often implicit—meaning that researchers’ assumptions and preferred theories and methods are hidden—as are the paradigms used in the preceding research, creating many layers of unacknowledged bias,^22^ often toward mainstream approaches and majority groups.^23^ In many branches of science, evidence is framed as a positivist endeavor, meaning knowledge already exists and humans simply have to discover it.^24^ This conflicts with approaches that argue that humans construct and frame knowledge according to assumptions and values and that these assumptions and values are hidden because they stem from existing structures and systems that often go unquestioned, such as capitalism, colonialism, and ableism.^25^ Public Health Critical Race praxis explains that many health researchers believe that scientific methods are objective, but a positivist paradigm biases research aims, interpretation, and dissemination and thus what is produced and considered knowledge.^23^

Although EIDM acknowledges that empirical evidence is context specific,^9^ it too often fails to acknowledge how culture and power inform which assumptions and values become embedded in what is considered useful evidence. For example, high school dropout prevention programs have evidence demonstrating how effectively they reduce labor productivity loss and crime and welfare costs.^26^ These programs, and many other social interventions, are often designed to be evaluated in economic terms that focus on individuals. Although social interventions have economic impacts, understanding interventions primarily through these lenses reinforces capitalist and individualist assumptions and limit the type of evidence being generated to understand whether and how such programs advance population health equity. For example, mental health interventions for individuals with mental health issues who are involved in the criminal justice system often use recidivism as a key success measure, giving less attention to improvement in mental health condition and other factors affecting recidivism.^27^ EIPM, which is a similar process to EIDM, is also often understood as a technical, value‐free or value‐neutral process undertaken by experts^28^—though many policy researchers and other scholars have argued that all parts of the policymaking process are inherently value driven and shaped by the people who engage with the process and their values.^28^, ^29^, ^30^

Health equity is a normative concept rooted in egalitarianism, the belief that all people are equal and should receive equal rights and opportunities.^31^ Health equity conceptualizes differences in health outcomes across populations as avoidable and unjust. These concepts of health equity, egalitarianism, and justice are value laden and can feel uncomfortable for researchers and policymakers to work within. However, to engage effectively with health equity, tools to assess evidence should acknowledge multiple ways of knowing, being, and doing driven by values within systems of power.^32^ As described by scholars collaborating with Indigenous communities in research, including and drawing from multiple knowledge systems can challenge positivist and other mainstream epistemologies as well as enrich evidence bases and align decision making and policymaking processes with concepts for fair and just decision making.^33^ We further recommend that those who generate, analyze, and use evidence examine assumptions and values implicit in evidence alongside explicit discussions of goals and values. This practice can shift EIDM and EIPM from upholding current structures of power and inequity and support their evolution. EIDM and EIPM can serve important functions in organizing, summarizing, and communicating possibilities for action to advance health equity if they acknowledge the implicit assumptions and values in evidence.

## Theoretical and Practical Considerations

In this section, we offer theoretical and practical considerations for use in employing EIDM to advance health equity within the constraints of the current evidence base.

### Understand Historical Context: Root Causes of Disparities and Inequity

Recent literature argues that those setting public health agendas—including identifying population health improvement priorities and targets, determining appropriate interventions, and securing funding and resources and overseeing their allocation—should not begin by looking at present‐day disparities but by looking at the roots of these disparities.^34^ Decision makers should ask themselves how and whether their agendas will change the systems that have historically oppressed some groups while distributing power and advantage to others.^34^, ^35^ For example, scholarship has established that present‐day health inequities in the United States are inseparable from multigenerational effects for those people who, racialized as Black, experienced race‐based slavery and were intentionally oppressed and defined as unequal by the state for hundreds of years.^34^ Such root cause understandings can reinforce a shift in public health interventions, away from individual behaviors and toward structural interventions, which focuses on the inequities perpetuated by institutions, people, and policies and the power structures associated with these.^36^

Foundational literature on fundamental cause theory from Link and colleagues explains desirable health outcomes as determined through fundamental causes, such as knowledge, money, power, prestige, and beneficial social connections,^37^ though many health models and frameworks since have focused on social conditions as the drivers of health.^35^, ^38^ More recent work by Heller and colleagues redirects attention to the structural determinants of health, defined as the following: “1) written and unwritten rules that create, maintain, or eliminate durable and hierarchical patterns of advantage among socially constructed groups in the conditions that affect health, and 2) the manifestation of power relations in that people and groups with more power based on current social structures work—implicitly and explicitly—to maintain their advantage by reinforcing or modifying these rules.”^13^ Many have clarified that the divergence in power and resources among groups, which leads to divergent health outcomes, is not inevitable but human created.^36^, ^39^ For example, the fact that unions could legally exclude individuals—a structural determinant—meant that those excluded had less access to jobs with union‐affiliated benefits, like sick leave. Those excluded, defined as belonging to particular racialized groups, were systematically deprived of access to jobs with more health‐supporting resources—leading to worse health outcomes.^38^

Evidence illustrating the positive impacts of historical structural change on population health can suggest how future interventions could impact health equity. Large structural changes’ effects on population health may be the best documented; for example, studies have found that the passage of the Civil Rights Act and abolition of Jim Crow laws decreased disparities in mortality between Black and White communities.^40^ Such findings prompt further investigation into root causes and structural change. Recent literature encourages scholars to look beyond health outcomes’ association with race and to measure racist practices and policies in US systems to better connect these structural determinants with health outcomes.^41^ Proposed measure areas include historical context (e.g., slavery), geographical context (e.g., the role of policing fines and fees in funding local budgets), and theory‐based novel quantitative and qualitative methods (e.g., measures of intersectionality).^42^ Recommendations for a measurement approach include “a national, publicly available data infrastructure on contextual measures of structural oppression,” with the overall aim being to expand understanding of structural forms of oppression “that are expressed as systematic exclusion of marginalized groups from power, resources, and opportunities within social, political, cultural, legal, and economic institutions.”^43^ Such efforts will better capture the impacts of future interventions and their relationship to structural solutions that address root causes. Scholars in this area have long pushed the population health field to reimagine what interventions might achieve health equity.

Some in population health have rightly concluded that both the roots of health inequity and the solutions to address that health inequity are understood.^16^ Researchers and practitioners can use processes to systematically evaluate whether their work addresses the root causes of inequities.^16^ For example, they can analyze the extent to which adult financial education programs can undo racial wealth gaps that are a cumulation of historical exploitation of assets in racially minoritized groups and what other interventions may be needed to close racial wealth gaps. They can assess how financial education programs guide participants to navigate structural barriers to equitable wealth‐building opportunities and acknowledge the strong relationship between income and health. We recommend that EIDM systematically account for historical context to better interrogate how and whether an intervention addresses the root, or fundamental, causes of health inequities. Such an evaluation can help decision makers understand whether an intervention will achieve its aims and may provide space for recalibration or redirection before an intervention is implemented. This practice can support researchers and practitioners in implementing interventions that change the systems that have distributed power and advantage to some groups while oppressing others.

### Understand Intervention Design and Intended Beneficiaries

Each type of intervention design—universal, targeted, and combined—has different advantages and disadvantages in terms of noneconomic and cost‐effective measures.^44^ Both universal policies and targeted policies can also influence inequalities through pre‐ or redistributive approaches; their effectiveness to address social disparities can vary by context.^45^ A review of public health policies in Australia shows how policies have different impacts on availability (in terms of place and time), affordability (regarding the cost of services), and acceptability (i.e., the capacity of services to meet various needs) of equity in access to programs and services by policy design and contextual features.^46^

#### Universal Interventions

Also called population‐level interventions, universal interventions are designed to benefit and improve overall conditions for all people in a population. Early scholars in universal interventions indicated that universal approaches could reduce disparities if the intervention reduces risk factors uniformly across the population and a group disproportionately experiences those risk factors.^47^ However, universal interventions do not guarantee equal effects for everyone or greater benefits for groups with higher health risk—the effects can vary by group characteristics and intervention context. For example, a review of universal school‐based health behavior interventions found that some groups benefit more than others, which led to inconsistent impacts on disparities in students’ drinking and smoking behavior.^48^ Universal interventions intended to improve health at the population level cannot reduce disparities unless they address fundamental root causes (e.g., racial residential segregation), and in fact, evidence suggests that they have the potential to increase disparities in outcomes.^49^, ^50^ Reviews of empirical research have shown that universal interventions’ effectiveness in reducing disparities varies by the level of intervention: Those that address systems and structures are more likely to decrease disparities, whereas those focused on individual behavior change are more likely to increase disparities.^48^, ^51^, ^52^, ^53^

#### Targeted Interventions

Targeted interventions are designed for population groups most impacted by specific disparities and enroll participants based on eligibility criteria such as income, health status, or neighborhood.^54^ One example is health and social care interventions for women and infants who experience social disadvantages or are from racially minoritized groups.^55^ Theoretically, such interventions can reduce disparities if the interventions are effective for the targeted groups and the outcomes for the nontargeted groups do not change.^56^ Based on such an assumption, evaluations of targeted interventions often measure absolute improvements for the targeted group and fail to further assess whether the intervention narrows or widens the gap between the targeted group and other groups.^57^ Moreover, although targeted individual‐focused interventions may be more effective to reduce disparities in health behavior than untargeted individual‐focused interventions,^58^ targeted interventions that lack an understanding of relevant structural factors and corresponding approaches in multiple domains (e.g., social, economic, physical environments, systemic discrimination, etc.) may have limited impact on social norms and environments that can sustain improvements in population health and health disparities.^59^

#### Interventions That Combine Universal and Targeted Approaches

Interventions that combine universal and targeted approaches are based on the understanding of strengths and weaknesses of each approach on a continuum of interventions.^54^ One combined approach, proportionate universalism, emerged in public health in recognition of a social gradient in health^60^—the consistent pattern between health status and socioeconomic status across the socioeconomic spectrum^54^—and is understood to address health inequities by both lowering the health gradient level and reducing health gaps.^61^ Proportionate universalism incorporates positive selectivism—providing some groups with additional services and resources proportionate to the level of disadvantage or needs—into universalism to offset structural disadvantages and flatten the health gradient.^60^ It also includes differentiation in the nature and delivery of interventions that are tailored to the needs of different groups (e.g., tailored services for different cultural groups).^60^ Examples of proportionate universal interventions are population‐based cancer screening programs and needs‐based geographical allocation of universal health care services in European countries.^56^ Even though proportionate universal interventions have a clear intention to effectively reduce health disparities and inequities while still enhancing population‐level health, there are challenges in planning and implementing such interventions, for example, difficulties in identifying and assessing needs of the marginalized group.^61^

We recommend that researchers and practitioners identify the intervention design—universal, targeted, or combined—as part of the EIDM process. Researchers and practitioners can analyze how universal interventions may reduce or exacerbate health inequities by disproportionately affecting some population groups who are at a greater risk of or more susceptible to unhealthy conditions (e.g., lead exposure, lack of green spaces in a neighborhood). Targeted interventions should be evaluated to assess their potential effectiveness in addressing the rights and unique needs of the underserved groups and mitigating systemic barriers that impede their health. Such design‐informed assessments can help uncover how an intervention may reduce health disparities in a population when empirical evidence is not available.

### Understand Differential Impact and Intersectionality

Generating evidence about an intervention's impact on equity does not come without complexities. It requires researchers and practitioners to shift from implementing interventions that have the potential to improve average health outcomes for the entire population to prioritizing interventions that improve overall outcomes and outcomes for the most impacted groups concurrently.^62^ It also requires researchers to collect and report on data that can describe any differential impacts an intervention has on groups, meaning how an intervention benefits some groups or areas more than others.^63^, ^64^ Employing EIDM requires a review of evidence with differential impact in mind. For behavior change‐focused universal interventions, different uptake of the interventions by individuals and groups can occur (partly because of their choice and aptitude) and result in differential impact on behavioral outcomes.^45^ In addition, in practice, not everyone has equal access to interventions that are designed to improve population health overall. For example, mandatory cancer screening at medical examinations and green‐space expansion benefit people covered by health insurance and wealthy neighborhood residents more, respectively.^51^, ^65^ Therefore, assessing a universal intervention's impact on disparities requires consideration of differential impact.

When analyzing differential impact, an oversimplified examination of disparities in outcomes among racialized groups can conceal disparities existing in other social domains (e.g., socioeconomic status, gender, sexual orientation, disability status) and within‐group heterogeneity. Examples include differences among racial minorities of an LGBTQ+ community, racially minoritized women, and immigrants with undocumented status. An intersectionality lens can lead EIDM to better interpretation and application of knowledge toward health equity. By incorporating intersectionality theory into population health research, researchers account for interactions among identity, social position, and processes of oppression or privilege of an individual or a group to examine disparities at various angles and recognize heterogeneity of disparity impact on populations.^66^ For example, studies on interventions to reduce homelessness among women should be grounded in an understanding of racialized, classed, and gendered systems and structural oppression that lead women to experience homelessness.^67^ These studies should also give implications for developing economic and housing policies that create secure, nondiscriminatory systems.

In practice, researchers often encounter challenges when examining diverse social identities and positions. These challenges include inconsistent measurements of social identities^68^ and small sample sizes that can limit the statistical power of within‐group comparisons.^69^ To address these practical issues, researchers should carefully consider sample size and accurate measurements based on theories and the existing empirical finding during the research design and data collection phases. Choosing the appropriate scale of equity measurement—between relative scale measures (i.e., risk or rate ratios) and additive (or absolute) scale measures (i.e., risk or rate differences)—presents another challenge. When the overall prevalence of a disease is low, relative disparities can be substantial, whereas additive disparities are reduced. Relative and additive disparity measures can also change in opposite directions when the prevalence of a disease within one group is very low relative to another. This highlights the importance of careful interpretation of disparity changes across different scales, as they may not always align.^70^

We recommend that researchers and practitioners consider how people with different social identities are impacted by an intervention in the EIDM process. This practice can help shift the EIDM process from prioritizing interventions that help the most people to prioritizing interventions that can benefit people with overlooked and undervalued social identities. The specific dimensions on which to measure equity are a key consideration in improving EIDM.

### Understand Community Context Before Implementing or Scaling Interventions

Employing EIDM to advance health equity requires considering how contributors and barriers to equitable implementation and outcomes operate layered social, cultural, and political contexts. This requires an assessment of the community context, including the following: actors involved in promoting or opposing strategies for health equity, networks, or relationships among key collaborators and prevailing ideas about local policy and who deserves service.^71^ Blending implementation strategies based on the community context—such as community needs and assets—can increase reach of health interventions for groups at higher risk of poor health outcomes.^72^

Understanding the community context before implementation—who is at risk, who has the most needs, which resources and infrastructures are available, who has been involved in or excluded from decision‐making processes—is the first step in replicating an evidence‐informed intervention in communities like or unlike the research setting. This is especially critical for public health agencies and policymakers in working with groups who experience marginalization. The Native American Center for Excellence emphasizes awareness of a Native nation's history (including historical trauma) and culture as well as community engagement in work with Native nations.^73^ According to DuMont and colleagues, “Equitable implementation occurs when strong equity components (including explicit attention to the culture, history, values, and needs of the community) are integrated into the principles and tools of implementation science to facilitate quality implementation of effective programs for a specific community or group of communities.”^74^ Equitable implementations include the following: 1) developing and tailoring strategies for implementation that explicitly consider disparities and contextual conditions, 2) generating practice‐based evidence through studying real‐world projects/experiments, and 3) engaging community organizations in both internal and external equity‐building efforts.^75^

Replicating and scaling evidence‐informed interventions are also challenged and mediated by implementation fidelity.^76^ Threats to implementation fidelity (for example, in enacting health policies) include failure to understand community or recipient needs, inadequate staff and financial resources, missing clarity on guidelines or responsibilities for implementation, missing organizational partnerships, conflicts with other policies, lack of political will, and societal stigma.^77^ Interventions implemented without attention to these points can worsen health inequities, especially when an intervention is replicated in a community context different from the one in which the intervention was evaluated.

We recommend that EIDM processes draw attention to community context that may modify an intervention's effects on health equity (e.g., community resources, values, partnerships). This practice can help ensure that researchers and practitioners can implement interventions in a way that maximizes their potential impact on reducing inequities. Table 1 illustrates how the synthesized approach can be used to evaluate the equity impact of baby bonds and healthy school lunch initiatives.

**Table 1:** Application of the Synthesized Approach to Evaluate Intervention's Equity Impact

Intervention	Historical Context	Intervention Design	Differential Impact	Community Context
Baby bonds	Forced relocation and genocide of Native communities; exploitation of slave labor; discriminatory wealth‐building opportunities^78^	Universal (for every newborn child) or targeted (for families with more financial support needs)	Families with lower incomes; racially minoritized families; Native families^79^, ^80^	Local or state program design and funding; stakeholder participation in design and implementation; program regulations about access and use of accounts
Healthy school lunch initiatives	Funding cuts to the National School Lunch Program budget; interest group influence on school nutrition standards^81^	Universal (for all students in a school or school district)	Families with lower incomes^82^	Stakeholders with power in school lunch decisions; barriers to healthy eating in students; funding sources

## Discussion

A synthesized approach to assess population health interventions for their potential impact on equity and disparities is possible. Although this must be complemented by attention to the inadequacy of the current evidence base to produce health for all in a society fraught with power imbalances, a synthesized approach would provide practitioners and policymakers with the tools to select and present information relevant to health equity. It could help focus interventions, such as programs and policies, to address existing disparities and ultimately shift power imbalances and the systems perpetuating such disparities. Without a synthesized approach, EIDM risks upholding current disparities and entrenching power structures that limit progress toward health equity.

Evidence clearinghouses, or evidence‐based program registries that provide curated research findings on policies or programs, are one of the ways that policymakers, practitioners, and others can access evidence to support EIDM. An opportunity exists for evidence clearinghouses, alongside other evidence translation work, to lead the way by evolving evidence analysis methods to critically assess the flaws of the current evidence base while also identifying the gaps in our understanding about how to effectively advance health equity. Evidence clearinghouses are uniquely positioned to contextualize and disseminate existing research findings to broader audiences. This likely includes expanding and evolving clearinghouse methods to explicitly name equity and frame evidence interpretation through an equity lens. The Community Guide's additional focus of systematic reviews on health equity relevance, Maternal and Child Health Innovations Database's equity‐focused assessment criteria, and What Works for Health's equity analysis are examples of clearinghouses’ efforts that lead the way.^21^

The opportunity also remains for individual researchers to design research questions, measurements, data collection, and analyses that are equity focused and intersectionality based.^78^ Researchers who seek to study how interventions can shift social structures should consider the matrix of power and oppression and the relevant social identities and positionality at play, then design their interventions accordingly.^79^, ^80^ In addition, an intersectional approach helps researchers and practitioners understand how systems of power and oppression are interwoven, reinforce each other, and sustain health inequities^81^, ^82^, ^83^ and can help researchers develop intersectionality‐grounded studies.^84^ Heller and colleagues recommended public health organizations and practitioners find, within the field's health promotion work, opportunities to build community power—“the ability of communities most impacted by structural inequity to develop, sustain, and grow an organized base of people who act together.”^85^ Researchers can partner with the community and generate evidence together to advance transformative narratives that promote equity, build power, and shift structures.

One potential complication is that this approach will take place in a complex and heavily politicized landscape. Among academics and policymakers, understanding evidence as neutral and authoritative may be appealing because it can be perceived as apolitical. Some researchers may be uncomfortable explicitly naming and centering a values‐driven approach in their work. Introducing explicit values may also be understood by some to undermine the legitimacy and credibility of evidence as a tool for decision making. However, evidence and EIDM processes are already challenged by an information environment filled with mis‐ and disinformation. In the United States, researchers and policymakers are often deemed “elites,” and their use of evidence may be viewed by communities with suspicion—US public trust in major societal institutions is historically low.^86^ Many also argue that this lack of trust from those in Native Nations and minoritized groups remains warranted, noting that government and institutional actions in settler colonial states continue to oppress and cause harm, that using data in decision making does not inherently address injustice,^87^ and that data practices must be intentionally inclusionary to avoid the “double moral injury” of health inequities and failing to see those inequities represented in data and addressed with resources.^88^ In this context, evidence may not be seen as salient, legitimate, or credible among much of the population. This suggests that those involved in research and EIDM processes could build public trust by acknowledging that evidence has always existed within and been shaped by a values‐driven political landscape and further arguing that selecting different values now is necessary to produce equitable health outcomes on competitive par with other countries. This also applies to values‐driven measurement considerations. For example, different measurements for disparities and inequity, such as relative or absolute disparity measures are values embedded. Furthermore, judgments about the most appropriate measure are also values laden, requiring careful consideration of the pros and cons of each approach.^70^

Other countries already actively use values‐driven frameworks to structure policy. For example, the UN Sustainable Development Goals (SDGs) call for “peace and prosperity for people and the planet,” and goals include an end to poverty, reduced inequality, and responsible consumption and production.^89^ These types of positive values, despite oppositional contexts, continue to endure—perhaps more so in countries where their broader policy adoption contributes to comparatively better health outcomes than in the United States, which often champions different values within a capitalist system. Research comparing Nordic countries and Australia suggest that the SDGs and Health in All Policies can be adopted in policy contexts in which the underlying values are less embraced, though more slowly and with a pragmatic approach that includes concessions.^28^ Further attention to values in the EIDM process in the United States and use of the theoretical and practical considerations offered in this paper can similarly contribute to a more constructive set of tools to advance health equity.

## Funding/Support

This work was supported by the University of Wisconsin Population Health Institute and the Robert Wood Johnson Foundation. The views expressed here do not necessarily reflect the views of the institute or the foundation.

## Conflict of Interest Disclosures

No disclosures were reported.
